# The Orbitofrontal Cortex Underlies Consolidation of Pavlovian Anticipatory Arousal Responses in Macaque Monkeys

**DOI:** 10.1523/JNEUROSCI.0619-25.2025

**Published:** 2025-07-28

**Authors:** Jaewon Hwang, Pamela L. Noble, Mary K. L. Baldwin, Elisabeth A. Murray

**Affiliations:** Section on the Neurobiology of Learning and Memory, Laboratory of Neuropsychology, National Institute of Mental Health, National Institutes of Health, Bethesda, Maryland 20892

**Keywords:** anhedonia, anthropoid, major depressive disorder, pavlovian learning, prefrontal cortex, reinforcer, reward

## Abstract

Altered arousal is characteristic of many mental health disorders, including major depressive disorder (MDD). Several studies link neural activity in the orbitofrontal cortex (OFC) with anticipation of reward, including anticipatory sympathetic arousal, which is blunted in MDD. We therefore studied acquisition and consolidation of appetitive pavlovian memories in two groups of adult male rhesus monkeys: unoperated controls (*N* = 4) and those with selective neurotoxic lesions of OFC (*N* = 4). The dependent measure was conditioned sympathetic arousal as indexed by differential pupil dilation, and the key comparison was dilation in response to a visual cue that predicted the delivery of a large fluid reward (CS+) versus a cue that predicted no reward (CS−). Control procedures ruled out global effects of the lesion on pupil dilation. All four unoperated controls and all four monkeys with OFC lesions acquired a conditioned increase in the pupil size in response to the CS+. However, three of the four monkeys in the lesion group failed to consolidate the memory underlying this response. In contrast to this impairment, monkeys with OFC lesions acquired and consolidated an operant visual discrimination for the same reward and did so at the same rate as controls. These findings point to a specialized role of OFC in consolidating memories underlying positive affective responses, which further implicates OFC dysfunction in the blunted positive affect characteristic of MDD and suggests therapeutic approaches involving enhanced consolidation and/or reconsolidation of associative memories.

## Significance Statement

Blunted anticipation of positive events is characteristic of many mental health disorders, including major depressive disorder. Cortical circuits underlying anticipatory responses have been identified in rodent models, but it is likely that different mechanisms operate in anthropoid primates, the clade that includes humans and monkeys. Anthropoids have cortical areas that rats and mice lack, and here we show that—in a macaque monkey model—some of these areas are necessary for consolidating memories that produce autonomic arousal in anticipation of rewards. Specifically, the integrity of granular orbitofrontal cortex (OFC)—the part of OFC specific to primates—is essential for macaque monkeys to consistently generate arousal in response to visual cues that predict positive events.

## Introduction

Anthropoid primates exhibit an impressive visual learning ability (Gaffan, 1994; Ghazizadeh et al., 2018), curiosity (Monosov, 2024), and a striking capacity for manipulating their environment (Almecija and Sherwood, 2017; Sobinov and Bensmaia, 2021). As a result, operant tasks have dominated studies of associative learning in these species (Schrier et al., 1965; Tomasello and Call, 1997). Autonomic responses, which occur in response to most positively and negatively valenced events, are much less studied despite the fact that they contribute to affective experience and behavior in a variety of settings.

Early studies involving electrical stimulation of the macaque cerebral cortex revealed a ring of cortical areas that, when stimulated, led to alterations in blood pressure, pupil diameter, and respiration, among other autonomic responses (Kaada et al., 1949 ; Sachs et al., 1949). Many of these areas—especially those in the orbital and medial frontal cortex—have been studied in the context of arousal and conditioned arousal. In marmosets, reversible, pharmacological activation of area 25—a medial frontal agranular area—has pronounced effects on arousal, altering the sympathetic-to-parasympathetic balance (Alexander et al., 2020). Activation of area 25 in marmosets yields increased arousal during threat extinction and recall (Alexander et al., 2020). In addition, manipulations of area 25 in both marmosets and macaques alter conditioned, or anticipatory, arousal in appetitive settings (Rudebeck et al., 2014; Alexander et al., 2019).

Manipulations of the orbitofrontal cortex (OFC) likewise influence arousal, although in different ways than manipulations of medial frontal areas such as area 25. Studies targeting subdivisions of OFC have revealed that reversible inactivation of anterior OFC (area 11) yields blunted conditioned arousal to conditioned stimuli (CSs) that predict either threat (exposure to darkness plus white noise) or reward (highly valued food), with no effect on arousal induced by unconditioned stimuli (USs; Stawicka et al., 2022). In contrast, inactivation of medial OFC (area 14) produces increased conditioned arousal to cues predicting a desired food (Stawicka et al., 2020).

In humans, several studies have pointed to a role of orbital and medial sectors of the frontal cortex (often referred to as the ventromedial prefrontal cortex or vmPFC) in modulation of arousal (Tranel and Damasio, 1994; Bechara et al., 2000; Sturm et al., 2018), including conditioned autonomic arousal that occurs in both appetitive and threat conditioning (Fullana et al., 2016; Harrison et al., 2017; Battaglia et al., 2020). The granular OFC (area 11 and granular parts of areas 13 and 14), in particular, is strongly implicated in prediction, evaluation, and comparison of appetitive outcomes in humans and monkeys. Importantly, these granular OFC areas have no homologs in the major rodent models, rats, and mice (Preuss and Goldman-Rakic, 1991 ; Preuss and Wise, 2022). Furthermore, several findings in humans link granular OFC with anticipation of reward (Gottfried et al., 2003; Howard et al., 2015), including a lowered anticipation of reward (anticipatory anhedonia) in patients with major depressive disorder (Xie et al., 2021), in youth at risk of depression (McCabe et al., 2012), and in individuals with a genetic risk of psychiatric disease (Ward et al., 2019 ).

Accordingly, we examined whether the granular OFC areas are essential for either the initial acquisition or consolidation of autonomic correlates of reward anticipation. Specifically, we tested the effects of neurotoxic lesions of granular OFC areas on the acquisition and consolidation of an appetitive pavlovian trace-conditioning task, using changes in pupil diameter as our measure of both initial learning and consolidation (Rudebeck et al., 2014). Although pupil diameter is determined by the activity balance of sympathetic and parasympathetic autonomic nervous system innervation of the iris dilator and iris sphincter muscles, respectively, the affective component of pupil size change in response to valenced images is likely driven via sympathetic arousal (Bradley et al., 2017). To narrow the interpretation of results, we also assessed pupil responses to unsignaled reward and changes in luminance, as well as the acquisition and consolidation of an operant visual discrimination.

## Materials and Methods

### Subjects

Eight adult male rhesus monkeys (*Macaca mulatta*), weighing between 8.3 and 12.7 kg at the beginning of the study, were used. Before training, four monkeys received bilateral, excitotoxic lesions of OFC, as described below. The other four monkeys served as unoperated controls and were tested concurrently with the operated group. Five of the eight monkeys, three operated (OFC 1–3) and two controls (Con 1 and 2), were the same monkeys studied for their decision-making abilities in earlier work from this laboratory (Rudebeck et al., 2017). The remaining monkeys were naive. All subjects were housed individually in a temperature- and humidity-controlled room on a 12 h light/dark cycle (lights on at 6:00 A.M.), and testing occurred during the light period. During the study, the monkeys were given controlled access to water to ensure sufficient motivation to respond in the test the apparatus. All procedures were reviewed and approved by the National Institute of Mental Health Animal Care and Use Committee.

### Surgery

Surgery was carried out in two stages, one hemisphere at a time, with at least 2 weeks intervening. Standard aseptic surgical procedures were used. Monkeys were immobilized with ketamine (10 mg/kg; i.m.) and then maintained with isoflurane gas (1–3% to effect). Throughout surgery, we monitored vital signs including blood pressure, respiratory rate, heart rate, body temperature, blood oxygen saturation, and exhaled/inhaled CO_2_. Each monkey received a series of injections of excitotoxins into the orbital cortex, described below, intended to produce loss of all neuronal cell types in all layers while sparing white matter projections passing nearby or through OFC. After the operation was completed, the scalp was closed in anatomical layers. All monkeys received a preoperative and postoperative treatment regimen consisting of dexamethasone sodium phosphate (0.4 mg/kg) and cefazolin antibiotic (15 mg/kg) for 1 d before surgery and 1 week after surgery to reduce swelling and to prevent infection, respectively. They also received Banamine (flunixin meglumine, 5 mg) for 3 d after surgery as an analgesic.

**Figure 1.**
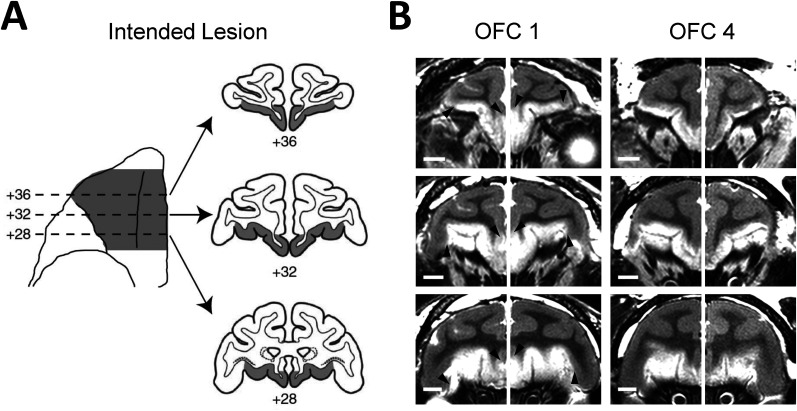
Excitotoxic lesions of OFC. ***A***, Intended extent of the OFC lesion (shaded region) shown on the ventral view of a macaque brain (left) and coronal sections through the frontal lobe (right). Numerals indicate the anterior–posterior distance from the interaural plane (0). Adapted from left panel of Figure 1 in Rudebeck et al. (2013). ***B***, Coronal images at corresponding levels taken from T2-weighted MRI scans obtained 3–4 d after surgery in two representative cases. White hypersignal is associated with edema and cell death that follows injections of excitotoxins. Left and right sides of the images are from different scans. Black arrowheads in OFC 1 point to edges of presumed lesion. Scale bar, 5 mm.

Under isoflurane anesthesia, a large bone flap was raised over the region of the prefrontal cortex, and a dura flap was reflected toward the orbit to allow access to the orbital surface. For the neurotoxic lesion, a series of injections was made into the cortex corresponding approximately to Walker's area 11, and the rostral, granular parts of areas 13 and 14 (Walker, 1940) using a handheld Hamilton syringe with a 30-gauge needle. Injections were made into the cortex on the orbital and medial surfaces of the frontal lobe between the fundus of the lateral orbital sulcus, laterally, and the rostral sulcus on the medial surface of the hemisphere, medially. At each site, 1.0 µl of ibotenate (10–15 µg/µl; Sigma-Aldrich or Tocris Bioscience) or a cocktail of ibotenate and *N*-methyl-D-aspartate (NMDA) (ibotenate 10 µg/µl, NMDA 10 µg/µl; Sigma-Aldrich) was injected into the cortex as a bolus. The needle was then held in place for 2–3 s to allow the toxin to diffuse into the tissue. Injections were spaced ∼2 mm apart, and the number of injections per hemisphere ranged from 86 to 119 (mean, 103). The location and extent of the intended lesion is shown in Figure 1*A*. Before training began, monkeys underwent an additional surgery to implant a titanium head post to allow stabilization of head position. Anesthesia and physiological monitoring procedures were the same as those used for the surgeries involving injection of excitotoxins. Monkeys recovered for an additional 30–40 d after the implant surgery.

### Apparatus

Training took place in a sound- and light-attenuating chamber (Crist Instrument) into which a primate chair was wheeled and secured. Monkeys were continuously monitored via an infrared camera. A fan mounted on the ceiling of the chamber provided ventilation and masked external noise. Stimuli were presented on a 19-inch monitor (1,024 × 768 pixels) that was positioned on the back wall of the chamber 45 cm in front of the monkey. Fluid rewards were dispensed through a sipper tube with volume controlled through a solenoid valve (Mitz, 2005). The solenoid was calibrated to deliver specific reward volumes that were associated with specific stimuli, as dictated by the experimental design. An infrared eye-tracking system (Arrington Research) was used to record gaze continuously during training and to record the pupil size as a measure of autonomic arousal (Mitz et al., 2017). Task control and data collection were carried out with the NIMH MonkeyLogic software (Hwang et al., 2019).

### Lesion assessment

The site of the neurotoxic lesion was confirmed with T2-weighted MR scans acquired ∼4 d after each surgery (Fig. 1*B*). In the T2 scans, white hypersignal reflects edema from the injections, and in cortical regions this typically reflects the extent of the lesion (Malkova et al., 2001; Basile et al., 2017). The location and extent of the white hypersignal were traced manually onto coronal sections of a standard rhesus monkey brain at 1 mm intervals, and then the volume of the affected area was calculated by summing the extent of the OFC covered by hypersignal across the anterior–posterior levels of the OFC.

The lesions were essentially as intended. Monkeys in the lesion group sustained substantial, bilaterally symmetrical damage to granular OFC, with minimal involvement of adjacent cortical areas, including the agranular OFC and the agranular insular cortex. The estimated volume of the OFC lesions averaged 80% (range, 59–94). Data for individual cases are provided in Table 1. All monkeys had slight inadvertent sparing of the cortex on the medial surface of the hemisphere immediately ventral to the rostral sulcus (area 14/10m), and in one monkey (OFC 4), there was substantial bilateral sparing of medial OFC area 14. Two monkeys (OFC 2 and OFC 4) had incomplete lesions of OFC due to bilaterally symmetrical sparing of the cortex in and near the fundus of the medial orbital sulcus. Another operated monkey (OFC 1) had slight bilateral sparing of the lateral portion of area 11. Finally, in two monkeys (OFC 1 and OFC 3), the injections of excitotoxin affected the cortex in the ventral portion of the frontal polar cortex rostral to OFC.

**Table 1.** Percentage estimated damage to OFC

Monkey	Left	Right	Mean
OFC 1	86.6	92.2	89.4
OFC 2	78.5	78.6	78.6
OFC 3	92.0	96.7	94.3
OFC 4	57.4	60.7	59.0

OFC lesion cases 1–4. Monkeys received excitotoxic lesions targeting Walker's areas 11, 13, and 14, bilaterally. Left, left hemisphere; Right, right hemisphere. Mean, the average estimated damage to OFC in the left and right hemispheres.

### Behavioral training

Monkeys performed a dual fixation–pavlovian conditioning task. In the full design, each test session comprised a relatively small number of pavlovian conditioning trials superimposed onto operant fixation trials, with the two tasks running independently. This was the same task used in a prior study (Rudebeck et al., 2014), with the exception that here we used fewer pavlovian trials per session. Training took place in two stages: the fixation-only task followed by the dual fixation–pavlovian conditioning task.

#### Fixation task training

**Figure 2.**
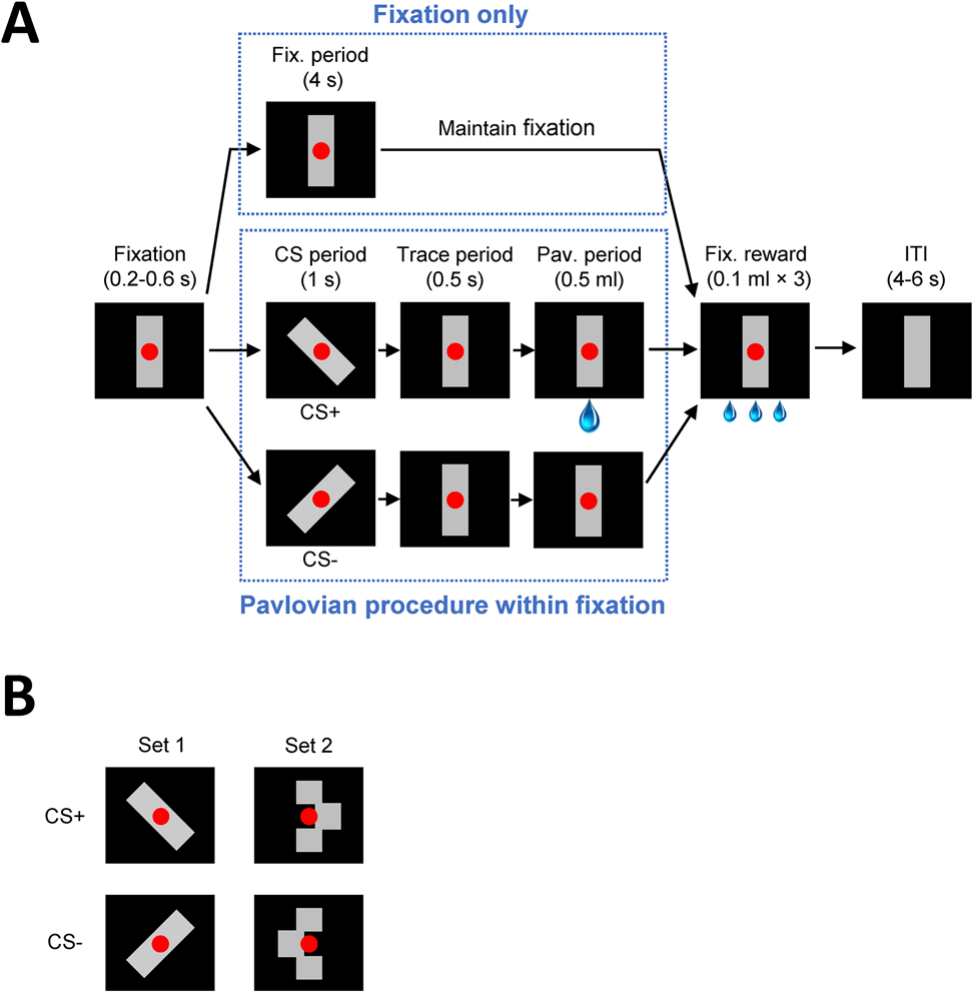
Dual fixation–pavlovian conditioning task. ***A***, Trial sequences of fixation and conditioning trials. Monkeys were required to maintain their gaze on the red spot for 4 s to receive three small drops of water. On a subset of trials, CSs, either CS+ or CS−, were presented for 1 s during either the 4 s fixation period or the ITI. If the CS+ was presented, a large drop of water (the US) was delivered 0.5 s after the offset of the stimulus. If the CS− was presented, no water was delivered. For analysis of the pupil size, only those pavlovian trials falling within the fixation period were considered. ***B***, CS sets. All stimuli contain the same number of gray pixels as the neutral stimulus (the vertical bar in ***A***).

Monkeys were first trained to fixate a central red spot (0.2° of visual angle) on a monitor screen for 4 s to earn a fluid reward (three drops of 0.1 ml water). Once they performed the fixation to a criterion of >80% completed trials for four consecutive sessions, a neutral stimulus (vertical gray rectangle) was introduced for habituation (Fig. 2*A*). The neutral stimulus was presented behind the red spot in the center of the screen, and monkeys were tested for 3 additional days to ensure stable and accurate performance. Monkeys were trained for 200 trials per session, one session per day.

#### Dual fixation–pavlovian conditioning: acquisition

Once the monkeys were reliably completing >80% of trials in the fixation task with the neutral stimulus, they were tested with the dual fixation–pavlovian conditioning task. Monkeys were still required to fixate the central red spot for 4 s on every trial, but now, in a subset of randomly selected trials, a pavlovian conditioning procedure was superimposed (Fig. 2*A*, lower blue dotted line box). The CSs could occur in either the fixation period or ITI. After a randomly selected, variable delay of 200–600 ms either the CS+ or CS− stimulus was presented for 1,000 ms. The CS then reverted to the neutral stimulus, a 500 ms trace interval ensued, and a 0.5 ml fluid reward was delivered (CS+), or no reward was delivered (CS−). The monkeys were trained at the rate of 200 trials per session, one session per day. Of the 200 trials, ∼32% (64 trials) involved a CS presented during fixation, and ∼12.5% (25 trials) involved a CS presentation during the intertrial interval. The CS+ and CS− appeared with equal probability. Pupil diameter was analyzed for all pavlovian trials falling inside successfully completed fixation trials. Training continued until each monkey exhibited a significant difference in the pupil size for the CS+ versus the CS− during the CS presentation periods for 4 consecutive days or for ∼50 sessions, at which time the monkey was considered to have failed the task.

Monkeys were tested with two sets of pavlovian CSs: Stimulus Sets 1 and 2 (Fig. 2*B*). Monkeys were first tested with Stimulus Set 1, in which the CS+ and CS− stimuli consisted of the gray neutral stimulus rotated 45° clockwise and counterclockwise. Assignment of CS+ and CS− to the two rotation directions was balanced within and across groups. When the neutral stimulus was altered to present either the CS+ or CS−, the same number of gray pixels was physically present on the screen throughout pavlovian conditioning trials, minimizing differences in luminance. This was done so that any within-trial alterations in the pupil size would most likely reflect changes in autonomic arousal and not simply changes associated with turning on or off the CSs or luminance differences between the CS+ and CS−. The dual fixation–pavlovian conditioning task was repeated with Stimulus Set 2. In Stimulus Set 2, the middle block of the neutral stimulus was shifted horizontally either to the left or the right to produce the CS+ and CS− (Fig. 2*B*). At least 7 d intervened between training with Stimulus Sets 1 and 2.

#### Dual fixation–pavlovian conditioning: extinction

After monkeys exhibited significant conditioned pupil dilation for 4 consecutive days, we conducted an extinction procedure. Monkeys were tested for two additional sessions, one per day, using the same task as before, but neither the CS+ nor the CS− led to reward delivery. Extinction sessions were conducted for both Stimulus Sets 1 and 2.

#### Unsignaled reward task

**Figure 3.**
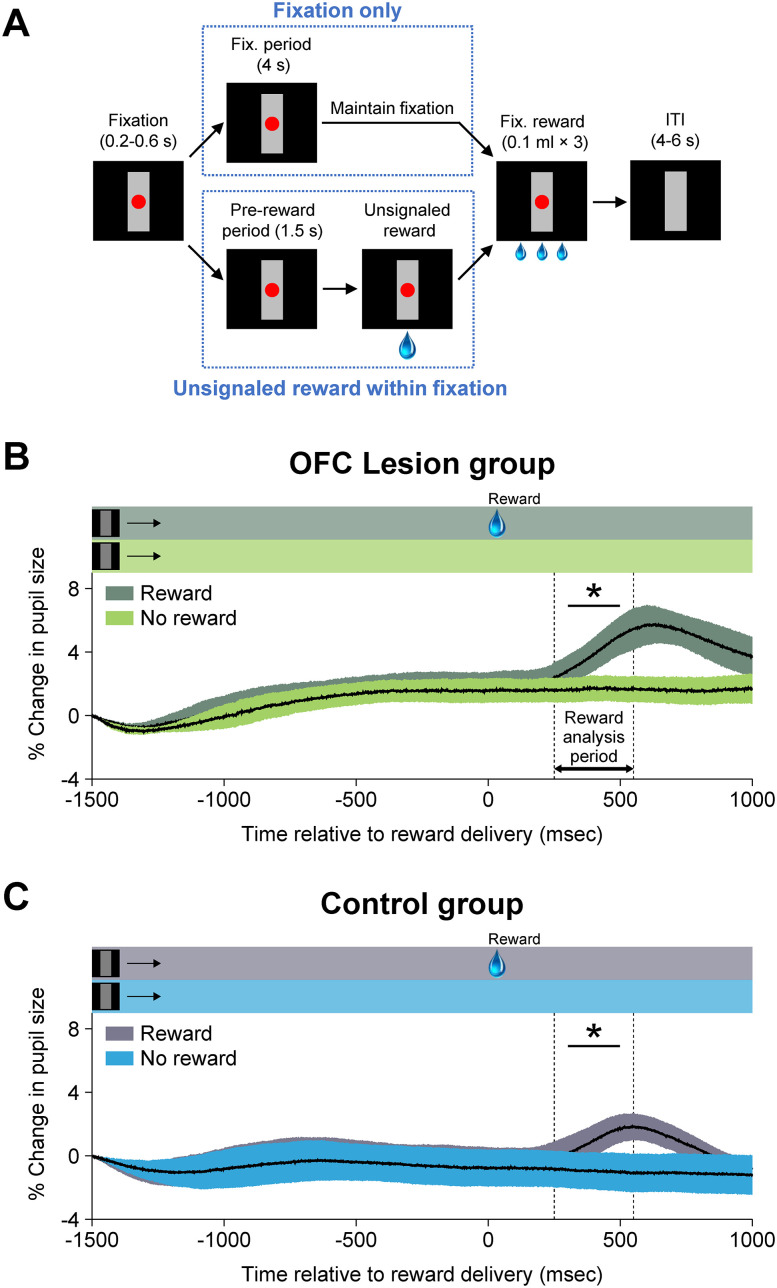
Unsignaled reward task and pupil size changes in response to unsignaled reward. ***A***, Monkeys were required to maintain their gaze on the red spot for 4 s to receive three small drops of water. On a small proportion of trials, a large drop of water (0.5 ml) was delivered during either the fixation period or the ITI, with no prior indication. For analysis of the pupil size, only those free rewards falling within the fixation period were considered. ***B***, ***C***, Pupil dilation is shown as a function of time (percent change, mean ± SEM) for the OFC lesion group (***B***) and control group (***C***). Shaded horizontal bars at the top of each panel show the timing of within-trial task events for each condition. Significance markers are based on the results of the ANOVA fitted for each group (the main effect of reward; see Materials and Methods). Shaded regions around curves indicate SEM. *p < 0.05. OFC lesion group, four rhesus monkeys with bilateral excitotoxic lesions of OFC areas 11, 13, and 14. Control group, four unoperated rhesus monkeys.

The same general task design and trial structure were used to provide unsignaled rewards. During these sessions, the same parameters were used as for the dual fixation–pavlovian conditioning task, but neither the CS+ nor CS− appeared (Fig. 3*A*). Instead, unsignaled fluid rewards (0.5 ml) were delivered with the same frequency and timing as pavlovian rewards had been delivered in the main task. Monkeys were tested for 4 consecutive days, 200 fixation trials per daily session.

#### Luminance test

**Figure 4.**
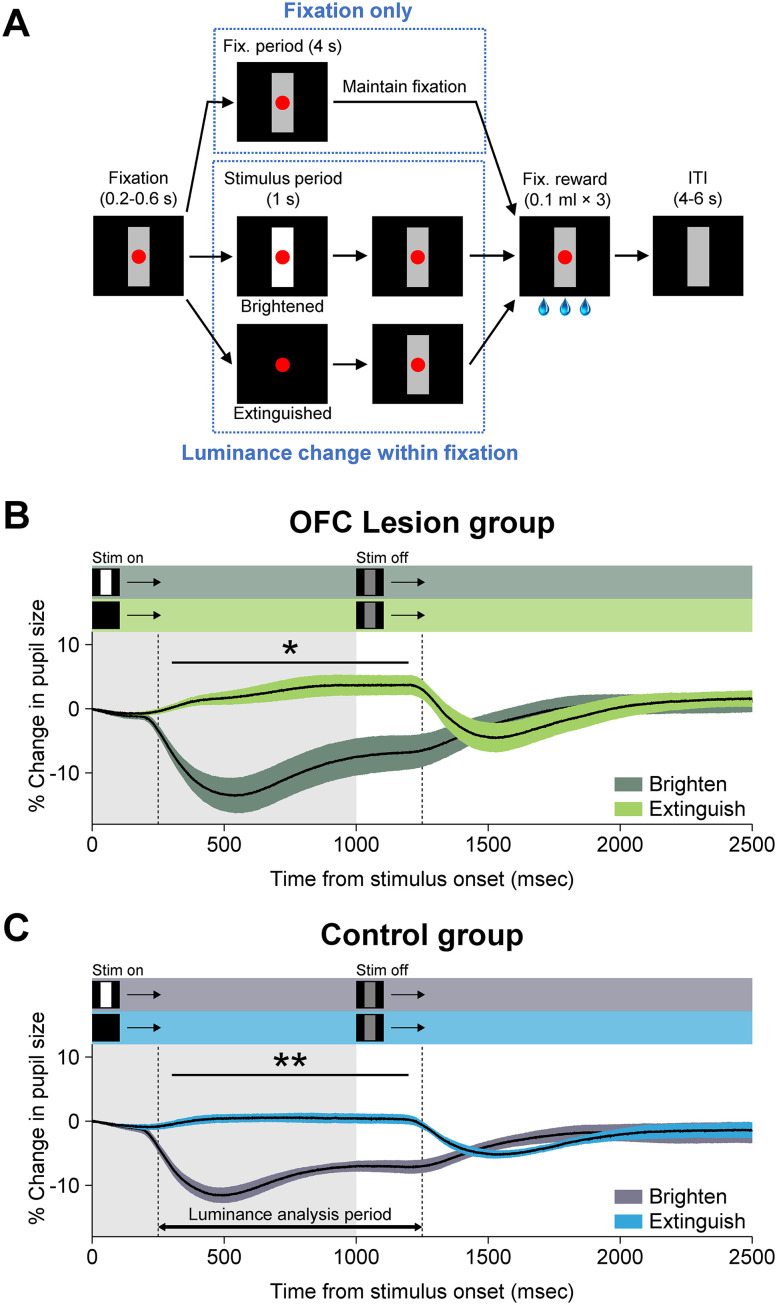
Luminance test and pupil size responses to changes in luminance. ***A***, Monkeys were required to maintain their gaze on the red spot for 4 s to receive three small drops of water. On a small proportion of trials, the gray vertical rectangle (neutral stimulus) was either brightened or extinguished for 1 s (stimulus period) during either the 4 s fixation period or the ITI. For analysis of the pupil size, only those luminance changes falling within the fixation period were considered. ***B***, ***C***, Mean ± SEM time course of percentage change in the pupil size following the brightening or extinguishing of the neutral stimulus is shown for the OFC lesion group (***B***) and control group (***C***). Shaded horizontal bars at the top of each panel show the timing of within-trial task events for each condition. Stim on, onset of increased (Brighten) or decreased (Extinguish) luminance of vertical gray bar. Stim off, return to original luminance of vertical gray bar. Significance markers are based on the results of the ANOVA fitted for each group (the main effect of stimulus type; see Materials and Methods). The gray-shaded region shows the duration of luminance change. Shaded regions around curves indicate SEM. **p < 0.01; *p < 0.05. OFC lesion group, four rhesus monkeys with bilateral excitotoxic lesions of OFC areas 11, 13, and 14. Control group, four unoperated rhesus monkeys.

The effect of varying luminance on extent and latency of monkey's pupil size responses was assessed in a separate task. Monkeys were required to fixate a central spot for fluid rewards (3 × 0.1 ml). Over the course of a session, instead of introducing a CS+ or CS−, we either extinguished the neutral stimulus or brightened the neutral stimulus for 1 s (Fig. 4*A*). Extinguishing or brightening the neutral stimulus occurred randomly and was not associated with the delivery of additional fluid reward. Matching the previous tasks, extinguishing or brightening of the neutral stimulus could occur either during the fixation period or the ITI with the same probability of CS+ and CS− stimuli in the main task. Monkeys were tested for 3 consecutive days, 200 fixation trials per daily session.

#### Operant visual discrimination task

**Figure 5.**
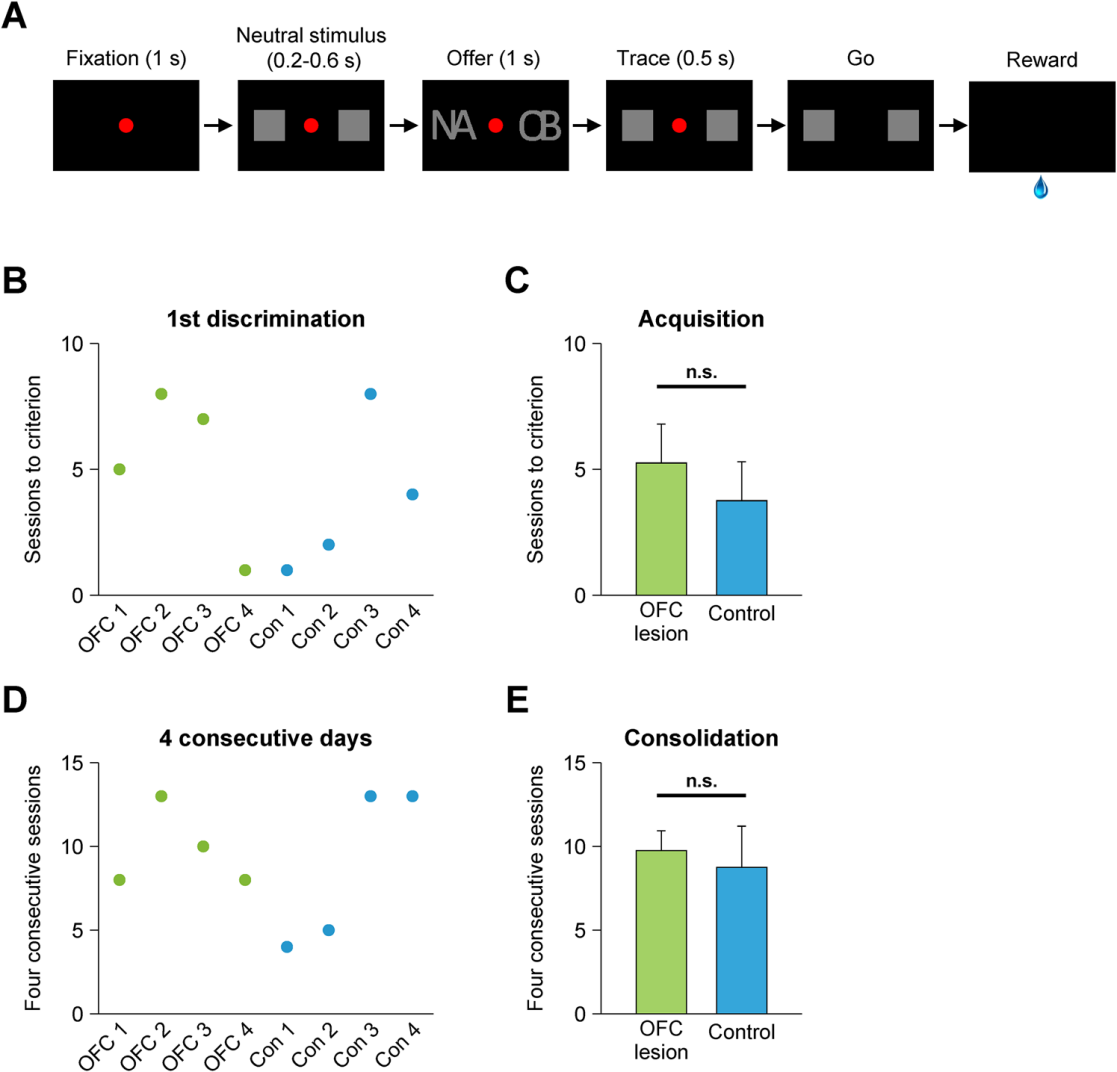
Visual discrimination task and acquisition of visual discriminations. ***A***, Monkeys were required to maintain their gaze on the red spot until it disappeared. While they maintained fixation, two neutral stimuli (gray squares) were presented on the left and right sides of the monitor screen. During the offer period, the neutral stimuli were switched to a pair of complex visual stimuli for 1 s: one item of the pair was associated with reward (S+) and the other was not (S−). Disappearance of the fixation spot served as the go signal; monkeys indicated their choice by making a saccade to either the left or right neutral stimulus. If the monkeys chose the neutral stimulus on the side of the screen on which the S+ had appeared, they received water (0.5 ml). The monkeys needed to learn through trial and error which stimuli were associated with reward (S+). To make the task modestly challenging, monkeys were required to learn two pairs of visual stimuli at a time. Trials using the two different pairs were interleaved. ***B***, The number of sessions required for each monkey to attain the criterion of above-chance–level performance for a single session. ***C***, Group mean sessions to criterion (acquisition) for the operant choice task. ***D***, The number of sessions required for each monkey to achieve criterion performance for four consecutive sessions. ***E***, The group mean number of sessions required to achieve consolidation, i.e., four consecutive sessions with above-chance–level performance. Error bars indicate SEM. n.s., not significant.

As a control, we assessed the ability of each monkey to acquire an operant visual discrimination response using stimulus material (e.g., grayscale images) and timing parameters similar to those used in the pavlovian conditioning procedure (Fig. 5*A*). Each trial began when the monkeys fixed their gaze on a central red spot for 1 s. While the fixation was maintained, two neutral stimuli were presented on the left and right sides of the screen, respectively. After a randomly selected, variable delay of 200–600 ms, the neutral stimuli were replaced with a pair of choice stimuli. Each stimulus was comprised of two grayscale ASCII characters, adjoined to create a single “object.” One stimulus of the pair was associated with reward (S+) and the other was not (S−). The choice stimuli were presented for 1 s and then reverted to the neutral stimulus. After a 0.5-delay period, the central red spot extinguished, which served as a signal that the monkeys could now make a choice via a saccade to either the left or right neutral stimulus. If the position of the chosen neutral stimulus was the same as that of the S+ on that trial, a fluid reward (0.5 ml) was delivered. The identity of the S+ was initially novel, so the monkeys were required to learn through trial and error. By design, the durations of presentation of the neutral stimuli, the discriminanda, and the delay period in the choice task matched the durations of the neutral stimuli, CSs, and the trace period in the dual fixation–pavlovian conditioning task. To make the task modestly challenging, two visual discrimination problems were administered concurrently. Thus, two sets of discriminanda were used for the entire training period, and they were interleaved trial by trial in a daily session. Monkeys were trained in each daily session until they earned 200 rewards or stopped of their own accord. In practice, they earned 146.2 ± 24.7 rewards (mean ± SD) per daily session. Training continued until each monkey chose the position of the S+ significantly more often than expected by chance (tested with binomial tests) for 4 consecutive days.

### Experimental design and statistical analyses

Pupil size signals were low-pass filtered at 100 Hz, sampled at 1 kHz, and then downsampled to 50 Hz for analysis. To avoid noise due to eye movements, only trials in which monkeys successfully maintained the gaze fixation for the duration of the fixation period (4 s) and the events of interest (i.e., CS+/CS− presentation, unsignaled reward, brightness change, etc.) occurred within the fixation period were included for the analysis of the pupil response. Blink artifacts were detected and removed with an in-house algorithm, and the gaps were linearly interpolated (Mitz et al., 2017). The pupil signals were baseline-corrected to a 100 ms period, −50 to 50 ms from CS onset, and normalized to control for the differences of the dynamic ranges among the subjects. Specifically, the mean pupil size measured for the 100-ms baseline period was subtracted from the entire pupil signal in each trial, and then the resulting pupil data were divided by the mean pupil size again.

The acquisition of the conditioned pupil response during the dual fixation–pavlovian conditioning task was evaluated for each session. Initial acquisition was reflected by the emergence of a differential pupil response to the CSs. We compared pupil responses across a 2,500 ms window (125 samples) starting from the onset of the CSs. Data were compared at each sample point using *t* tests, and the results were Bonferroni-corrected for multiple comparisons (*p* < 0.0004). Acquisition of a conditioned response was deemed successful when a monkey demonstrated significantly different pupil response to the CS+ and CS− at more than five sample points during the CS period within a single session. This screening procedure was conducted for each session until monkeys achieved four consecutive sessions with differential pupil responses to the CS+ versus CS− during the CS period, our criterion for consolidation of the conditioned association.

Pupil response data from the 4 consecutive days of the criterion run were analyzed together using a mixed-design ANOVA. To account for the slow change in the pupil size following an event, analysis periods were shifted 250 ms forward in time. Pupil size changes to the CSs were analyzed separately for three nonoverlapping periods: the CS period, 250–1,250 ms after the CS onset; the trace period, 1,250–1,750 ms after the CS onset; and the reward period, 1,750–2,050 ms after the CS onset. The ANOVA model included within-subject factors of the stimulus set (Set 1, Set 2), CS (CS+, CS−), and session. A variable for each individual monkey was included as a random factor nested under the between-subject factor, group (lesion, control). On occasion, for further analysis, this model was fitted for each group separately after the group variable was removed.

Data from the unsignaled reward task and the luminance test were analyzed with a similar model. This model included one between-subject factor, group, and two within-subject factors, stimulus type (reward vs no reward or brightened vs extinguished, depending on the task) and session. A variable for each monkey was included as a random variable and nested under group. For further analysis, this model was also fitted for each group separately without the group variable.

## Results

Monkeys were trained on a task in which pavlovian conditioning of stimulus–reward associations was superimposed on operant conditioning of active visual fixation (Fig. 2*A*). The pavlovian conditioning procedure and the operant fixation task proceeded independently. Therefore, if a monkey broke fixation when a CS was presented during the fixation period, the fixation spot was extinguished, but the pavlovian conditioning continued unaffected by the oculomotor behavior. As training proceeded, we expected the monkeys to exhibit an increased pupil size in response to the CS+ compared with the CS−, as observed in a previous study using this task (Rudebeck et al., 2014).

### Acquisition and consolidation

**Figure 6.**
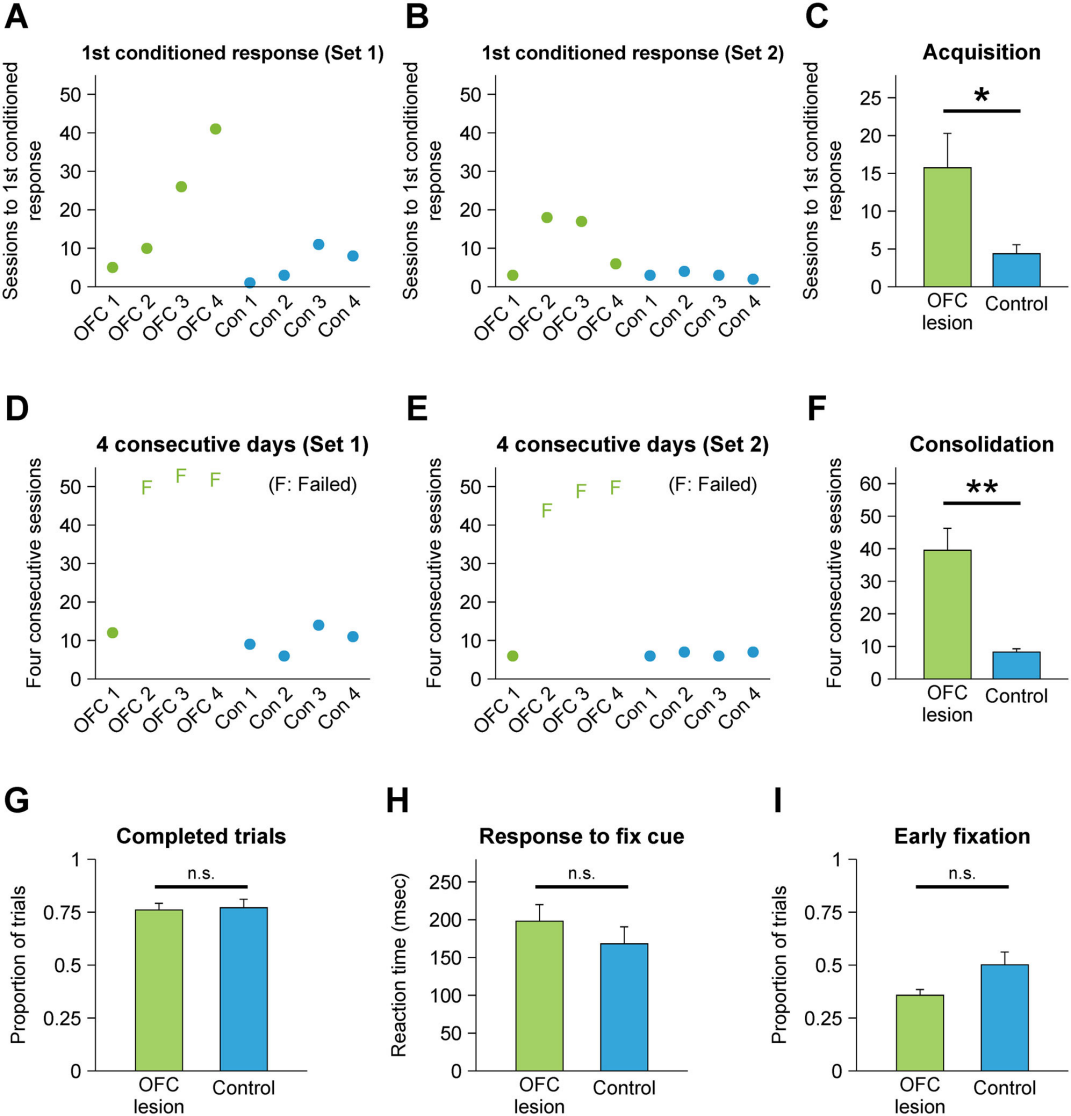
Behavioral performance during the dual fixation–pavlovian conditioning task. ***A***,***B***, The number of training sessions required for each monkey to acquire a significant conditioned autonomic response is shown for Stimulus Set 1 (***A***) and Set 2 (***B***). ***C***, The group mean number of sessions to acquire the first conditioned response. Data for the two stimulus sets are combined. ***D***,***E***, The number of sessions required for each monkey to maintain the conditioned autonomic response for four consecutive sessions is shown for Stimulus Set 1 (***D***) and Set 2 (***E***). Three monkeys in the lesion group failed to maintain the response for four consecutive days (F) in both stimulus sets. ***F***, The group mean number of sessions required to achieve consolidation, i.e., four consecutive sessions with conditioned autonomic responses. The two stimulus sets are combined. For those monkeys that did not show the conditioned response for 4 d, the total number of their training sessions is used instead. Training was suspended after monkeys completed ∼50 sessions. ***G***, Mean proportions of successfully completed trials for each group during all training sessions. ***H***, Mean reaction time for monkeys to acquire the central gaze fixation after the fixation cue was presented. Those trials in which monkeys were looking at the center even before the fixation cue presentation are shown in ***I*** and not included in this analysis. ***I***, Mean proportions of trials in which monkeys fixated on the center of the screen before the fixation cue was presented. Error bars indicate SEM; **p < 0.01; *p < 0.05; n.s., not significant.

**Figure 7.**
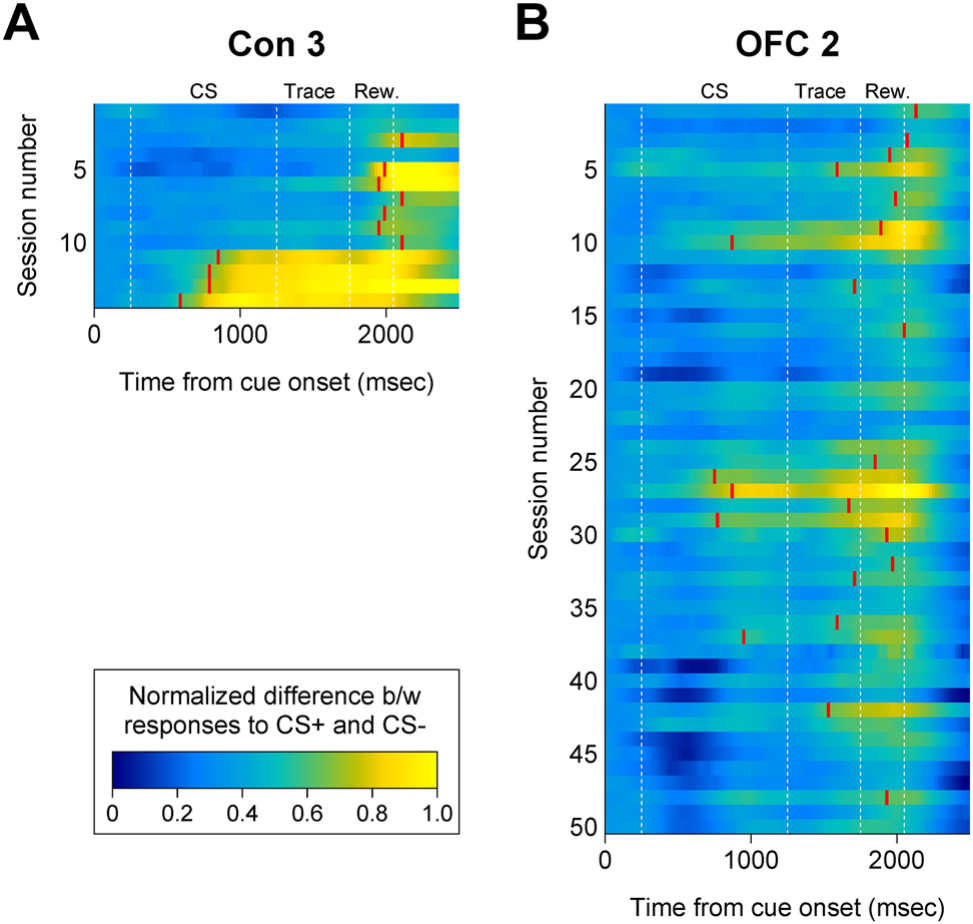
Acquisition of conditioned pupil dilation across training sessions. ***A***, ***B***, Data from representative monkeys in the control (Con 3) and OFC lesion (OFC 2) groups. Each row corresponds to one session and shows the difference in the pupillary response to CS+ versus CS− in that session. The red line indicates the first bin in which the difference became significant. At the beginning of training, the significant difference mostly occurred in the reward-analysis period (Rew.). As the training proceeded, however, the difference began to occur during the CS-analysis period. The control subject maintained this early CS period pupil dilation for 4 consecutive days, but the lesion subject was unable to do so. Dashed vertical lines demarcate the analysis periods: CS, CS-analysis period; Trace, trace-analysis period; Rew., reward-analysis period.

As expected, monkeys readily acquired a conditioned autonomic response to the CS+ (Fig. 6*A*,*B*). The mean number of sessions to the first conditioned response was 15.8 ± 4.5 (mean ± SEM) for the OFC lesion group and 4.4 ± 1.2 for the controls (Mann–Whitney *U*, *p* < 0.05; Fig. 6*C*). Whereas the controls maintained the conditioned pupil response across days, it quickly became apparent that the monkeys with OFC lesions did not. To capture this phenomenon, we measured the number of sessions to attain the criterion of four consecutive sessions with a significant conditioned response (Fig. 6*D*,*E*), which signaled consolidation of the conditioned association. Controls typically required only three additional sessions beyond the first session of acquisition to achieve consolidation. In contrast, three of the four monkeys with OFC lesions failed to sustain this conditioned response across sessions, even when given an extended training period (49.7 sessions on average). During training, they were able to show a significant conditioned pupil response no more than 2 d in a row. Accordingly, the effect of the OFC lesion is best characterized as an impairment in consolidating appetitive pavlovian associations rather than a comprehensive impairment in acquisition. This pattern is clearly conveyed by an analysis that shows progress across sessions (Fig. 7). The fourth monkey in the OFC lesion group, labeled OFC 1 in Figure 6, acquired the pavlovian conditioned response in the same manner as the controls.

To determine whether the impairment in acquisition and consolidation of a conditioned pupil response could be accounted for by a low level of overall performance or lack of motivation, as opposed to an inability to acquire stimulus–outcome associations in our design, we compared three behavioral measures from the dual fixation–pavlovian conditioning task between the groups: proportion of completed fixation trials, reaction time to the fixation cue, and early fixation to the fixation cue. We assumed that greater motivation would be indexed by fewer aborted trials, shorter latencies to fixate the fixation spot at the beginning of trials, and a greater number of “early” fixations (e.g., gazing at central fixation even before the fixation cue was presented). We found no group differences in these three indicators (Fig. 6*G*–*I*) and conclude that the group differences in the acquisition and consolidation of the conditioned pupil response cannot be accounted for by global differences in performance or motivation that might have resulted from the lesion.

**Figure 8.**
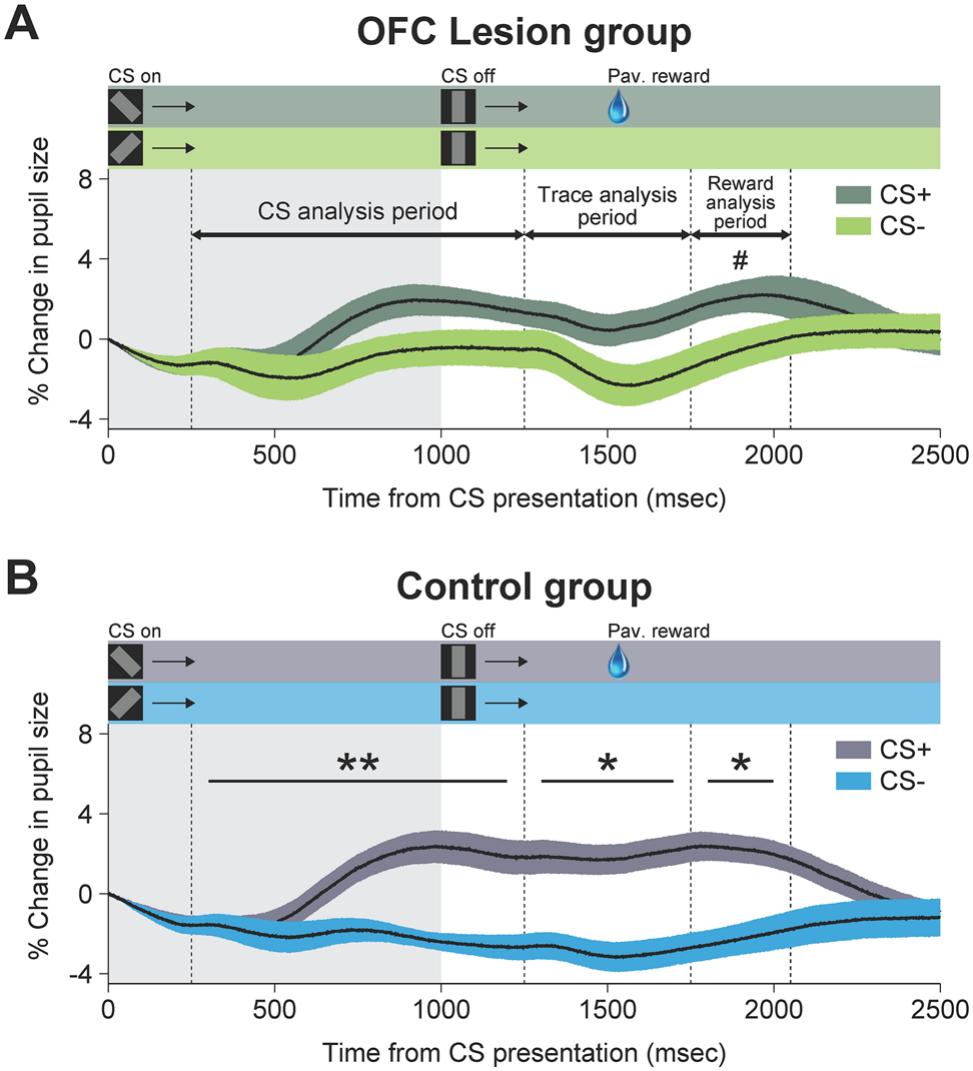
Conditioned autonomic response during the dual fixation–pavlovian conditioning task. ***A***, ***B***, Pupil dilation is shown as a function of time (percentage change, mean ± SEM) for the OFC lesion group (***A***) and control group (***B***). Shaded horizontal bars at the top of each panel show the timing of within-trial task events for each condition. Data from Stimulus Sets 1 and 2 are combined. Because the pupil response lags the trial events by ∼250 ms, each analysis period is shifted by 250 ms relative to the CS onset, offset, and reward delivery. Significance markers are based on the results of the ANOVA fitted for each group (the main effect of CS; see Materials and Methods). The gray-shaded region shows the duration of CS presentation. Shaded regions around curves indicate SEM. **p < 0.01; *p < 0.05; #, marginally significant (*p* ≈ 0.0589). OFC lesion group: four rhesus monkeys with bilateral excitotoxic lesions of OFC areas 11, 13, and 14. Control group: four unoperated rhesus monkeys.

Next, we analyzed the pupil size changes during trials. This analysis was conducted with data collected from the four consecutive sessions of criterion performance (i.e., consolidation sessions) for each monkey. For those monkeys that failed to sustain the conditioned pupil response across four sessions, we used four consecutive sessions from the first day on which they exhibited a significant conditioned response. The analysis was performed for the three task epochs separately: CS period, trace period, and reward period (Figs. 2*A*, 8). Note that due to the time required for the pupil response, the analysis periods are offset from the actual task epochs by 250 ms. As a result, the analyses reflect data collected in the three offset epochs: CS-analysis period, trace-analysis period, and reward-analysis period (Fig. 8). Overall the monkeys showed an increased pupil size on CS+ trials for each task epoch (main effect of CS, *F*_(1,6)_ = 16.12; *p* < 0.01 for the CS period; *F*_(1,6)_ = 19.90; *p* < 0.01 for the trace period; *F*_(1,6)_ = 18.93; *p* < 0.01 for the reward period). There was no main effect of group nor an interaction between the group and CS type. However, whereas the pupil dilation to the CS+ was significantly different from that to the CS− during all task epochs in the control group (main effect of CS, *F*_(1,3)_ = 44.63; *p* < 0.01 for the CS period; *F*_(1,3)_ = 16.78; *p* < 0.05 for the trace period; *F*_(1,3)_ = 10.64; *p* < 0.05 for the reward period), this was not the case for the lesion group. Monkeys with OFC lesions failed to show differential pupil responses during the CS and trace periods (main effect of CS, *F*_(1,3)_ = 2.46; *p* > 0.05 for the CS period; *F*_(1,3)_ = 4.65; *p* > 0.05 for the trace period), although they showed marginally significant pupil responses during the reward period (*F*_(1,3)_ = 8.83; *p* ≈ 0.0589). In other words, during the CS and trace periods, the monkeys with OFC lesions did not show the same increase in autonomic arousal exhibited by the controls. Thus, there are two main findings: (1) OFC is not necessary for acquisition of conditioned arousal in an appetitive pavlovian context; and (2) OFC is essential for consolidation of the conditioned associations that are acquired in an appetitive pavlovian context.

Considering all eight monkeys, our analysis did not reveal a significant interaction between the group and CS type. There are at least two possible reasons for this. First, there is a limit to the maximum size of pupil dilation, so our measures for the control group might be under the influence of a ceiling effect. Second, one of the monkeys with an OFC lesion (OFC 1) acquired a conditioned response at the same rate as the controls and displayed a change in the pupil size to the CS+ as great as that of the controls, so the group effect was countervailed to some extent. When we performed the same analysis after excluding this monkey, there was a significant interaction between the group and CS type for the CS period (*F*_(1,5)_ = 18.98; *p* < 0.01) and a marginally significant interaction for the trace period (*F*_(1,5)_ = 5.83; *p* ≈ 0.0604).

### Extinction

**Figure 9.**
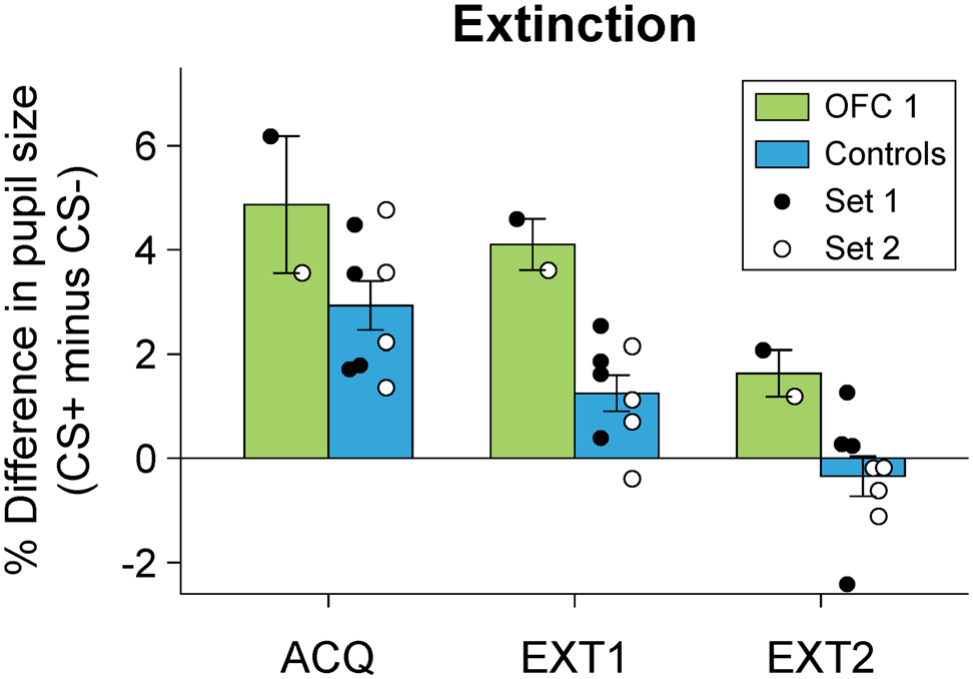
Mean (±SEM) difference in pupil responses during the CS period, CS+ minus CS−, during the final day of acquisition (ACQ) and two extinction sessions (EXT 1 and 2). Symbols represent the responses of individual monkeys (filled circles, Stimulus Set 1; Open Circles, Stimulus Set 2). Negative values indicate pupillary constriction. Control group: four unoperated rhesus monkeys.

To confirm that the conditioned increase in the pupil size was related to the positive emotional nature of the reward associated with CS+ and not another variable, such as stimulus identity, we tested the monkeys that attained criterion under extinction conditions for two additional sessions. During extinction, the CS stimuli were presented as in the main task, but no reward followed the CS+. Then we compared pupil responses for the last acquisition session and two extinction sessions with a mixed-design ANOVA (Fig. 9). As expected, the pupil size was smaller during extinction sessions relative to the last acquisition session for both the CS and trace periods (effect of the CS type, *F*_(1,3)_ = 80.83; *p* < 0.01 for the CS period; *F*_(1,3)_ = 62.37; *p* < 0.01 for the trace period; CS type × session interaction, *F*_(2,6)_ = 8.65; *p* < 0.05 for the CS period; *F*_(2,6)_ = 7.04; *p* < 0.05 for the trace period). The one monkey with an OFC lesion that attained criterion also showed this decrease to the same extent. By the second extinction session, the difference in pupil response to the CSs was abolished (Fig. 9). The fact that all monkeys extinguished responding to CS+ suggests that the previously observed increases in the pupil size were directly related to the positive nature of the reward associated with the CS+.

### Pupil response to unsignaled reward

The findings from the extinction sessions strongly suggest that the effect of the OFC lesion in the present task was limited to autonomic signaling of reward anticipation. In support of this idea, the OFC lesion group showed intact autonomic responses to the US, i.e., to receipt of a large fluid reward (Fig. 8). However, it is possible that the OFC lesion altered autonomic responses to the US outside the context of the task. To test this possibility, we ran an unsignaled reward task in which the same large drop (0.5 ml) of water was delivered without any prior indication (Fig. 3*A*). In this task, both groups showed significant increases in the pupil size after the delivery of unsignaled rewards, compared with the trials in which the reward was not delivered (effect of reward, *F*_(1,6)_ = 36.99; *p* < 0.001 for the reward period; Fig. 3*B*,*C*). Therefore, the diminished autonomic arousal during the CS and trace periods in the lesion group cannot be explained by the reduced arousal to the primary reinforcer.

### Pupil response to changes in luminance

Another possible explanation for our results is that damage to OFC produces a deficit in controlling the pupil size that is unrelated to reward. If that were true, the diminished pupil response in the monkeys with OFC lesions could be attributed to a generalized inability to regulate the pupil size. To rule out this explanation, we brightened or extinguished the neutral stimulus during the fixation period or the ITI, instead of presenting the CSs (Fig. 4*A*). The changes in the luminance of the neutral stimulus were not associated with the delivery of an additional reward. As expected, brightening or extinguishing the neutral stimulus changed the pupil size significantly during the luminance-analysis period (effect of stimulus type, *F*_(1,6)_ = 67.71; *p* < 0.001; Fig. 4*B*,*C*). However, there was no effect of the group (*F*_(1,6)_ = 0.13; *p* > 0.05) nor an interaction between the group and stimulus type (*F*_(1,6)_ = 0.24; *p* > 0.05), which indicates that both groups responded to the light change similarly and did not differ in the extent of pupil dilation or constriction.

### Operant visual discrimination

To confirm that lesions of OFC did not affect the ability of monkeys to learn stimulus–reward associations as assessed by explicit choice, we also tested our monkeys with a standard visual discrimination task (S+ vs S−) in which the stimulus presentation and delay parameters were chosen to match those used in the pavlovian task (Fig. 5*A*). Initially the identity of the reward-associated cue (S+) was unknown to the monkeys, so they needed to learn by trial and error. Trials with two stimulus sets were interleaved pseudorandomly, and monkeys were trained to criterion. The lesion group required 5.2 ± 1.5 d (mean ± SEM) until they chose the S+ significantly more frequently than the S− in a single session, and the control group took 3.8 ± 1.5, but this difference in acquisition was not statistically significant (Fig. 5*B*,*C*). Moreover, both groups were able to maintain the selection of the S+ for 4 consecutive days, thereby exhibiting consolidation, with no group difference in the number of total training sessions (Fig. 5*D*,*E*). Therefore, the failure to consolidate the appetitive pavlovian task by monkeys with OFC lesions is unlikely to be due to factors related to the stimulus type, the delay (trace) interval between stimulus presentation and reward, or the use of a neutral stimulus. Instead, it appears that OFC is essential for consolidating conditioned autonomic arousal to complex visual stimuli that predict positive emotional events.

## Discussion

We examined the acquisition and consolidation of appetitive pavlovian memories in macaques with neurotoxic lesions of granular OFC (areas 11, 13, and 14) and unoperated controls. Conditioned sympathetic arousal was assessed by differential pupil dilation in response to the CS+ versus CS−. All monkeys acquired a conditioned response to the CS+. Whereas controls rapidly consolidated the pavlovian associations, three of the four monkeys with damage to OFC failed to do so. Because all monkeys showed robust pupil diameter adjustments following unsignaled rewards and changes in luminance, we can rule out global effects of the lesion on pupil function. In addition, all monkeys learned an operant visual discrimination task using similar stimuli, times of stimulus exposure, and delay parameters. Thus, as expected from previous studies (Izquierdo et al., 2004; Rudebeck et al., 2017), OFC lesions disrupted neither visual perception nor the ability to acquire a rule. Accordingly, our results show that, in macaque monkeys, granular OFC is essential for consolidating pavlovian associations that mediate sympathetic autonomic arousal in anticipation of positive outcomes.

### Comparison with other OFC findings

Neurophysiological studies in macaques and functional imaging studies in humans have revealed that activity of neurons in OFC represent the sensory properties of foods and fluids, including odor, taste, flavor, temperature, and texture (see Murray et al., 2025 for review). While monkeys perform tasks involving choices between visual stimuli to earn different amounts and types of fluid reward, OFC neurons encode variables such as the reward type, magnitude, and probability at the time of stimulus presentation and reward receipt (Padoa-Schioppa and Assad, 2006; Kennerley et al., 2009; Stoll and Rudebeck, 2024). Thus, the activity of OFC neurons reflects features of both anticipated and received rewards.

In a study by Morrison and Salzman (2009), macaque monkeys learned and performed a pavlovian trace-conditioning task in which three visual CSs predicted one of three USs: a large fluid reward, a small fluid reward, or a mildly aversive air puff directed to the face. Some OFC neurons encoded both appetitive and aversive CSs, and others responded to primary reinforcers of all types, as well as to the CSs. Importantly, during learning, activity encoding the CSs paralleled acquisition of the conditioned responses (licking or blinking). These findings support the idea that OFC contributes to consolidating stimulus–outcome associations in both appetitive and aversive pavlovian settings.

Appetitive pavlovian conditioning is thought to result in behaviors elicited by cognitive representations of the outcomes (US) used in conditioning. Accordingly, one explanation of the consolidation impairment we observed is that the circuitry representing specific outcomes, which includes granular OFC, is missing, and this prevents the establishment of long-term associative memories involving these representations. The occurrence of within-session acquisition of conditioned arousal can be accounted for by associations established transiently in other circuits. For example, the basolateral amygdala, central nucleus of the amygdala, and perirhinal cortex all contribute to acquisition of pavlovian associations (Everitt et al., 2003; Bucci and Burwell, 2004; Braesicke et al., 2005; Balleine and Killcross, 2006), and these regions may be sufficient to support acquisition in the short term. However, the integrity of OFC is essential for both establishing and consolidating associations yielding reliable, predictive increases in arousal.

The release of dopamine in the ventral striatum by neurons of the ventral tegmental area (VTA) occurs during conditioning. In rats, OFC is thought to contribute to this process by signaling the value of an unexpected reward to VTA dopamine neurons, which in turn calculate reward-prediction errors (Takahashi et al., 2009). Although there is at best a sparse projection from OFC to VTA in macaques (Frankle et al., 2006), there could be an indirect route via the striatum or subthalamic nucleus. If granular OFC of macaques is responsible for signaling the reward value to VTA, either directly or indirectly, the loss of OFC could explain the consolidation impairment. In the absence of OFC, no mechanism could drive dopamine neurons in VTA to support acquisition and consolidation of the pavlovian associations. Equally plausible, however, is the possibility that OFC is essential for receiving dopaminergic signals from VTA, and this leads to establishment and consolidation of conditioned associations. Dopaminergic terminals are widespread in granular OFC (Brown et al., 1979 ; Lewis et al., 1988 ; Zubair et al., 2021), so OFC could (1) signal reward value to VTA to permit the generation of reward-prediction errors by VTA dopaminergic neurons and (2) receive teaching signals from VTA neurons that serve to consolidate newly acquired stimulus–outcome associations within OFC. Consistent with the foregoing, lesions of OFC (Pears et al., 2003) and dopamine depletion in OFC (Walker et al., 2009) of marmosets disrupt learning involving visual CS and auditory conditioned reinforcers.

In the context of pavlovian appetitive conditioning, reversible inactivation of anterior OFC area 11 but not posterior OFC area 13 of marmosets decreased arousal during the CS period (Stawicka et al., 2022). There was no effect on autonomic responses to the US. Notably, inactivation of area 11 in marmosets produced the same direction of effect as did OFC lesions in our case. The studies differ, however, in at least one important way beyond the temporary versus permanent nature of the lesion and the species studied. Whereas the marmosets had already acquired the pavlovian associations, we examined acquisition and consolidation. These results call for a systematic investigation of area 11's role in conditioned arousal for both learning and consolidation and for both appetitive and threat contexts.

### Comparison with medial frontal areas

The impairment in consolidation shown here contrasts with intact consolidation of the same associations by monkeys with aspiration lesions of the subgenual anterior cingulate cortex (sgACC; area 25; Rudebeck et al., 2014). Although monkeys with sgACC lesions acquired and consolidated a conditioned response as quickly as controls, their conditioned pupil dilation occurred in two separate periods: during the CS period and after delivery of the US. In unoperated controls in that study, as here, the conditioned response bridged the time gap between the two. These findings suggest a specialization of granular OFC for consolidating stimulus–outcome associations and for sgACC in sustaining arousal across time gaps and incorporating it into other behaviors.

### Comparison with findings in humans

These findings inform the prediction of positive emotional events in humans. A study of pavlovian threat conditioning found that, unlike healthy participants, patients with damage to vmPFC failed to exhibit conditioned skin conductance responses (SCR) during threat conditioning (Battaglia et al., 2020). Like our study, Battaglia and colleagues used visual stimuli as CSs, although we studied prediction of positive outcomes as opposed to negative ones. Taken together, and considering the findings of Stawicka et al. (2022), who showed that inactivation of anterior OFC in marmosets blunted conditioned arousal in both appetitive and aversive settings, these findings suggest that OFC may be responsible for reliably generating appropriate arousal to cues predicting both positive and negative outcomes.

Also relevant are results from the Iowa gambling task, in which participants draw cards from one of the four decks to earn money (Bechara et al., 1996). Although both decks contain cards that produce gains and losses, two of the decks yield net gains (advantageous), the two other net losses (disadvantageous). Participants could learn to make good choices by choosing cards from the advantageous decks. Unlike controls, patients with vmPFC damage failed to show SCR in anticipation of choices from disadvantageous decks. So, just as humans with vmPFC damage failed to generate anticipatory arousal to visual stimuli that predicted a new loss (Bechara et al., 1999), our monkeys with OFC lesions lacked anticipatory arousal to visual stimuli that predicted rewards, except for a transitory time during training.

Although our findings agree with those of Bechara et al. in some ways, there are discrepancies. When tested on a pavlovian task with visual CSs and loud noises as USs, some patients with vmPFC damage, like controls, developed anticipatory SCRs during the conditioning phase (Tranel et al., 1996; Bechara et al., 1999). Assuming that OFC is included in vmPFC, this seems to contradict our results. One possible explanation is that some patients with vmPFC lesions displayed acquisition but might not have continued to show conditioned SCR (i.e., consolidation) had they been tested for several days. Also, the task did not employ a discriminative CS design, and dependence on OFC may hinge on that factor or other methodological differences.

### Limitations

One of the monkeys with an OFC lesion consolidated learning normally. The lesion extent did not account for this finding (Table 1), but there was bilateral sparing of the cortex in lateral area 11. Interestingly, two other studies implicate area 11 in conditioned autonomic responses. Based on a lesion-voxel-symptom mapping approach, Battaglia et al. (2020) pointed to Brodmann's area 11 (corresponding approximately to medial frontal areas 10r and 11m of Ongur et al., 2003) as the likely source of impairment in acquisition of conditioned threat. Additionally, the findings from inactivation of anterior OFC in marmosets suggest a role for area 11 (Stawicka et al., 2022). Another possibility is that this monkey learned the stimulus–reward contingency via an alternative mechanism operating independently of OFC.
